# Recent cancer incidence trends in an observational clinical cohort of HIV-infected patients in the US, 2000 to 2011

**DOI:** 10.1186/1750-9378-8-18

**Published:** 2013-05-24

**Authors:** Elizabeth L Yanik, Kristen Tamburro, Joseph J Eron, Blossom Damania, Sonia Napravnik, Dirk P Dittmer

**Affiliations:** 1 Department of Epidemiology, University of North Carolina at Chapel Hill, 130 Mason Farm Rd, Chapel Hill, NC, USA; 2 Department of Microbiology and Immunology, University of North Carolina at Chapel Hill, 715 Mary Ellen Jones Building, Chapel Hill, NC, USA; 3 Division of Infectious Diseases, University of North Carolina at Chapel Hill, 130 Mason Farm Rd, Chapel Hill, NC, USA; 4 Department of Microbiology and Immunology, University of North Carolina at Chapel Hill, 31–353 Lineberger Comprehensive Cancer Center, Chapel Hill, NC, USA; 5 Division of Infectious Diseases, University of North Carolina at Chapel Hill, 130 Mason Farm Rd, Chapel Hill, NC, USA

**Keywords:** Kaposi sarcoma, AIDS, HIV, AIDS-associated malignancies, Cancer

## Abstract

**Background:**

In HIV-infected populations in developed countries, the most recent published cancer incidence trend analyses are only updated through 2008. We assessed changes in the distribution of cancer types and incidence trends among HIV-infected patients in North Carolina up until 2011.

**Methods:**

We linked the University of North Carolina Center for AIDS Research HIV Clinical Cohort, an observational clinical cohort of 3141 HIV-infected patients, with the North Carolina Cancer registry. Cancer incidence rates were estimated across calendar years from 2000 to 2011. The distribution of cancer types was described. Incidence trends were assessed with linear regression.

**Results:**

Across 15,022 person-years of follow-up, 202 cancers were identified (incidence rate per 100,000 person-years [IR]: 1345; 95% confidence interval [CI]: 1166, 1544). The majority of cancers were virus-related (61%), including Kaposi sarcoma (N = 32) (IR: 213; 95%CI: 146, 301), non-Hodgkin lymphoma (N = 34) (IR: 226; 95%CI: 157, 316), and anal cancer (N = 16) (IR: 107; 95%CI: 61, 173). Non-Hodgkin lymphoma was observed to decrease from 2000 to 2011 (decline of 15 cases per 100,000 person-years per calendar year, 95%CI: -27, -3). No other changes in incidence or changes in incidence trends were observed for other cancers (all P > 0.20).

**Conclusions:**

We observed a substantial burden of a variety of cancers in this population in the last decade. Kaposi sarcoma and non-Hodgkin lymphoma were consistently two of the greatest contributors to cancer burden across calendar time. Cancer rates appeared stable across calendar years, except for non-Hodgkin lymphoma, which appeared to decrease throughout the study period.

## Introduction

Among HIV-infected patients increased risk of cancer, such as Kaposi sarcoma (KS) and non-Hodgkin lymphoma (NHL), has been recognized since the beginning of the HIV epidemic [1,2]. This is believed to be a result of HIV-induced immune suppression hindering the control of cancer-associated viruses, as well as direct effects of HIV replication [3]. While KS and NHL still contribute substantially to morbidity, the spectrum of cancers seen in people living with HIV is changing rapidly [4]. Cancer registries have been extensively used to study these trends [5,6]. However, the most recent data from registries and prospective cohort studies has lagged behind by many years. The most recent US-based and Swiss-based cohort trend analyses end in 2006 and 2007 [7-9], while Globocan, the International Agency for Research in Cancer-based worldwide cancer registry, reports data up to 2008 [6].

We investigated cancer incidence trends in the University of North Carolina (UNC) CFAR HIV Clinical Cohort (UCHCC) up to 2011, hypothesizing that declining cancer incidence trends that marked the introduction of combination antiretroviral therapy (ART) may plateau in more recent years at a level still significantly elevated compared to the general population.

## Methods

### Study population

For this study we linked UCHCC study participants with North Carolina state cancer registry data between 2000 and 2011. The UCHCC is an observational clinical cohort which includes all HIV-infected patients who have received HIV primary care at UNC since 1996 [10]. The UCHCC includes demographic, laboratory, and diagnosis data, including thorough reviews and adjudications of all cancer diagnoses based on patient medical records. The North Carolina state cancer registry is also administered through UNC. This study was approved by the UNC Institutional Review Board. This research was not experimental, but observational in nature and was in compliance with the Helsinki Declaration.

#### Statistical analysis

Patients contributed time during all years in which they were in care at UCHCC, as indicated by clinic visits and the presence of at least one HIV RNA level or CD4 cell count result. Overall clinical and demographic characteristics were described weighted by person-time. Time-varying characteristics, such as age and laboratory values, were updated for each year. Laboratory values were based on the first measurement in a given year. For cancer cases, the laboratory values measured closest to the date of cancer diagnosis were used. Cancer incidence rates for each calendar year were calculated as the number of diagnoses divided by the number of person-years. If a patient was diagnosed with multiple cancers, all cancer diagnoses were counted.

To estimate absolute changes in cancer incidence across calendar time we used linear regression. Additionally, to test whether incidence trends were changing across calendar years for the most frequent cancer types we relied on broken stick regression [11]. Similar techniques have been used previously to uncover cancer trends in AIDS populations [12,13]. Broken stick regression fits two linear piecewise regression lines and determines the breakpoint at which the regression slope changes. Likelihood ratio tests (LRT) were used to evaluate improvements in model fit with one added breakpoint. All analyses were done in SAS version 9.2 and R version 2.15.1.

## Results

**Figure 1:**
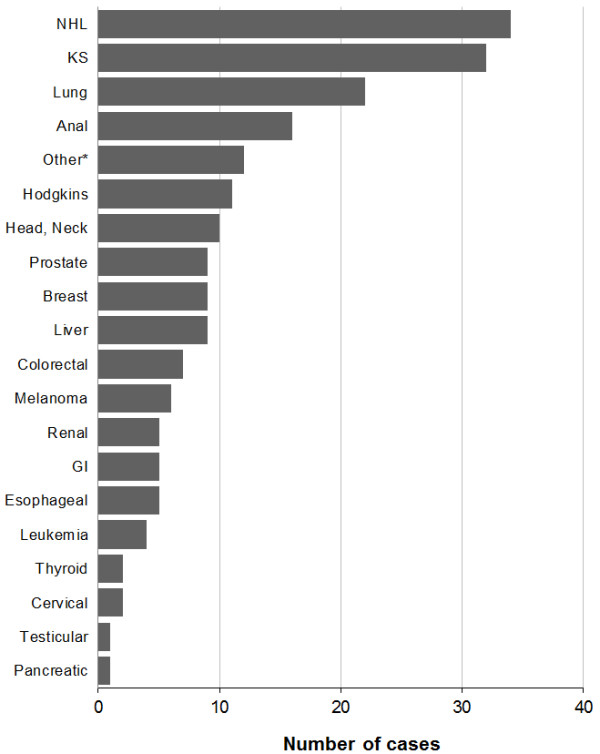
**Cumulative cancer counts in the University of North Carolina Center for AIDS Research HIV clinical cohort (2000 – 2011).** *Other includes vulvar cancer, multiple myeloma, throat cancer, unspecified lymphoma, and other unspecified malignancies. NHL = Non-Hodgkin Lymphoma, KS = Kaposi sarcoma, GI = Gastrointestinal.

From Jan. 1, 2000 through Aug. 1, 2011, 3141 HIV-infected patients attended at least one clinic visit, and contributed a total of 15,022 person-years of observation. We observed a total of 202 cancer cases in this study population, including a wide variety of cancer types (Figure 1). In absolute numbers of cases, KS (N = 32) and NHL (N = 34) were the most common cancers in this HIV-infected population. Among the human papillomavirus (HPV)-associated cancers, anal cancer was common (N = 16), while invasive cervical cancer was rare (N = 2). Lung cancer was the most frequent non-AIDS-defining cancer (NADC) (N = 22). As a group, NADCs were more frequent than AIDS-defining cancers (ADCs) (134 NADC cases vs. 68 ADC cases); however, individually each NADC cancer type was less common than either KS or NHL.

Overall 30% of patients were female, 59% were black, and 31% were white (Table 1). Over time, the median patient age was 43 (IQR: 36–50), the median CD4 count was 440 cells/mm^3^ (IQR: 244–660), and the median viral load was 2.4 log_10_ copies/ml (IQR: <1.7-4.1). Patient demographic and clinical characteristics changed across calendar years of this study. In more recent calendar years patients receiving HIV care were older, with higher CD4 cell counts and were more likely to have HIV RNA levels below the limit of detection (Table 1).

**Table 1:** Demographic and clinical characteristics of patients in the University of North Carolina Center for AIDS Research HIV Clinical Cohort, stratified by calendar year intervals 2000-2011

**Characteristic N (%)**	**2000-2002**	**2003-2005**	**2006-2008**	**2009-2011**	**Total**	P*
Total	1866	1849	1843	1767	3141	
Female sex	598 (31.8)	601 (32.3)	544 (29.3)	514 (28.4)	932 (29.7)	0.03
Race						
Black	1136 (61.0)	1088 (58.9)	1047 (57.0)	997 (56.4)	1862 (59.3)	
White	591 (31.7)	610 (33.0)	596 (32.3)	563 (31.9)	962 (30.6)	
Other	142 (7.5)	153 (8.2)	202 (10.9)	207 (11.7)	317 (10.1)	<0.01
MSM	566 (30.3)	623 (33.6)	640 (34.6)	516 (29.2)	889 (28.3)	<0.01
IDU	261 (14.0)	248 (13.4)	208 (11.2)	152 (8.6)	340 (10.8)	<0.01
Age (years)^†^	40 (34–46)	42 (36–49)	44 (37–51)	46 (38–52)	43 (36–50)	<0.01
CD4 cell count (cells/mm^3^) ^†^	376 (181–602)	403 (218–621)	440 (251–657)	519 (335–737)	440 (244–660)	<0.01
HIV RNA (log_10_ copies/ml) ^†^	2.92 (2.30-4.47)	2.73 (BLD-4.45)	2.05 (BLD-4.05)	BLD (BLD-2.58)	2.37 (BLD-4.11)	<0.01
ART experience (person-years after ART exposure)^‡^	3322 (81.6)	3505 (83.7)	3680 (83.8)	3871 (88.0)	14378 (84.3)	<0.01

*P-values from Chi-squared test for categorical and binary characteristics, and Kruskal-Willis test for continuous characteristics. Undetectable HIV RNA results were assigned a value of half the limit of detection for the purposes of assessing trends across time.

^†^Median (Interquartile Range) presented for continuous characteristics.

^‡^The number of years of follow-up within the time interval contributed by ART-experienced patients and the percentage of all follow-up within the time interval that was contributed be ART-experienced patients.

BLD: below limit of detection (<50 copies/mL); ART: antiretroviral therapy; MSM: men who have sex with men; IDU: injection drug user.

Among all cancer cases, the median CD4 count at cancer diagnosis was 244 cells/mm^3^ (IQR: 109–458), lower than the median CD4 count observed in the overall patient population. CD4 counts at diagnosis were lower for KS cases (median: 69 cells/mm^3^; IQR: 34–227) and NHL cases (median: 153 cells/mm^3^; IQR: 89–263), but were more similar to the entire patient population for other cancer cases (median: 370 cells/mm^3^; IQR: 159–582). Six of the 32 KS cases were the presenting diagnoses for HIV. Cancer cases had a median HIV RNA at cancer diagnosis of 2.3 log_10_ copies/ml (IQR: <1.7, 4.3) similar to the median of 2.4 log_10_ copies/ml observed in the overall patient population. However, it was higher in KS cases (median: 4.4 log_10_ copies/ml; IQR: 2.2, 5.2) and NHL cases (median: 3.4 log_10_ copies/ml; IQR: 2.6, 5.0), but lower in other cancer cases (median: <1.7 log_10_ copies/ml; IQR: <1.7, 2.9). When compared to the rest of the patient population, cancer cases were also more likely to be male (83% vs. 70%), men who have sex with men (40% vs. 35%), and older (median age 47 years vs. 43 years). Antiretroviral naïve patients contributed 16% of all follow-up time, while 21% of cancers were diagnosed in patients who were antiretroviral naive.

**Figure 2:**
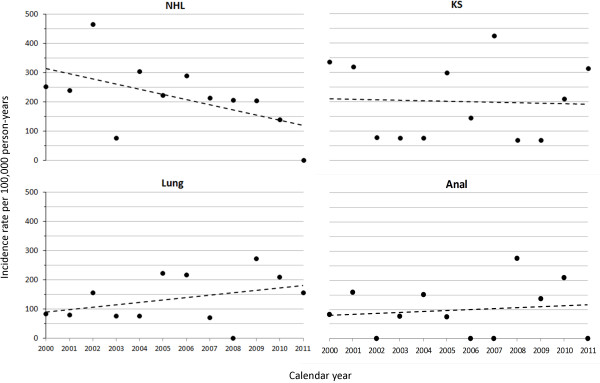
**Graph of selected cancer trends in the University of North Carolina Center for AIDS Research HIV clinical cohort (2000–2011).** Circles represent observed incidence rate in each calendar year. Dotted line represents trend over time estimated from linear regression. NHL = Non-Hodgkin lymphoma, KS = Kaposi sarcoma.

The overall incidence rate of NHL was 226 cases per 100,000 person-years (95%CI: 157, 316) but declined relatively consistently from 2000 through 2011 with a decline of 15 cases per 100,000 person-years per calendar year (95%CI: -27, -3) (Figure 2). While KS was the next most common cancer (IR: 213; 95%CI: 146, 301), a statistically significant increasing or decreasing trend was not found (2 cases per 100,000 person-years decrease per year, 95%CI: -22, 19). For lung cancer we observed an incidence rate of 146 cases per 100,000 person-years (95%CI: 92, 222). While lung cancer incidence appeared to increase over time, a significant trend was not identified (8 cases per 100,000 person-years increase per years, 95%CI: -5, 21). Anal cancer with an overall incidence of 107 cases per 100,000 person-years (95%CI: 61, 173) showed no clear changes in incidence over time. We did not observe incidence rate trends across calendar years for any of the other cancers considered (all P for trend > 0.20). Additionally, broken stick regression models did not demonstrate a statistically significant improvement in model fit, indicating the absence of detectable changes in cancer incidence trends over time. For most cancers, the number of cases was too small to discern incidence trends with no recorded cases in many calendar years.

## Discussion

We observed a substantial decline in NHL throughout the study period from 2000 to 2011. This demonstrates a continued decline from prior studies that showed decreasing NHL incidence up until 2006 [7,8,14]. Declines in prior studies were attributed to the introduction and dissemination of ART; while the continued decline in NHL we observe may be due to earlier initiation of effective ART as evidenced by the higher CD4 counts observed over time. Interestingly, a decreasing trend over calendar time was not evident for KS. Additionally, no clear trends over calendar time were identified for NADCs.

In our study, less than 20% of KS cases were the presenting diagnosis for HIV, while close to 30% of KS cases developed in patients with CD4 counts greater than 200 cells/mm^3^, and more than 20% developed in patients with undetectable HIV RNA. An interesting development in recent years has been the emergence of KS in subjects on successful long-term ART [15]. These subjects may be the reason why KS incidence in recent years remains significant and unlike NHL did not decline further. Our observation suggests that the damage done by initial HIV infection, ongoing immune activation [16], sub-optimal immune reconstitution [17] and perhaps long-term antiretroviral exposure continues to place HIV-infected individuals at increased risk for KS. Longer survival for HIV-infected patients may also allow more time for the emergence of KS. Viral cancers have a long lag time between primary infection and clinical disease. KS in HIV + patients manifests itself approximately seven years after seroconversion for KS-associated herpesvirus [18,19]. As such, the initiating event for cancer that manifests itself clinically in the early periods of widely available ART could have taken place when patients were exposed to less than optimal antiretroviral therapy or no therapy at all.

While a number of NADCs were observed in our cohort, incidence trends were not apparent for anal cancer or lung cancer, the most frequent types. A recent North American multi-site study of anal cancer including the UCHCC population also found no change in incidence between 2000–2003 and 2004–2007 [20]. Shiels et al. suggest that the clinical impression of more and more NADCs in recent years is largely due to the aging effect of the HIV + population [4]. The HIV population may face larger increases in cancer risk than the general population with advancing age due to more cumulative exposure to environmental carcinogens such as tobacco and alcohol. We urgently need to understand how anti-cancer regimens and ART drugs interact, in order to develop optimal treatment approaches. While the AIDS Malignancy Consortium is beginning to identify such approaches for NHL and KS, further studies will be needed to address similar concerns in treating NADCs in HIV-infected patients [21,22]. In contrast to the high incidence of anal cancer, few cases of cervical cancer were observed. This is in part because our patient population was disproportionately male, but also likely serves as an indicator of the success of efforts to screen and treat premalignant cervical lesions.

Our study is noteworthy because we included over 10 years of data spanning the most recent calendar years (2000–2011). During these years new antiretroviral agents and their combination were introduced with better tolerability and greater efficacy in controlling HIV replication [23,24]. The improved effectiveness of these agents is demonstrated in part by the higher CD4 cell counts and greater proportion of our study population with suppressed HIV RNA levels in more recent calendar years. Most prior studies assessing cancer incidence trends did not cover the most recent years [5,7] . Although our study focuses on a study population receiving care at one academic center, it represents an area of the US with an increasingly large burden of HIV, including a large proportion of women (almost one-third), and large percentage of African American patients. Also close to a third of this cohort resides in rural areas [25]. Our observations reflect these aspects of the changing HIV population [26,27].

These data do not take into account recent evidence supporting the benefits of earlier ART initiation and the subsequent changes in HIV treatment recommendations in the U.S [23,28,29]. It is likely that as these new recommendations are phased in, the spectrum of malignancies affecting HIV-infected individuals will change. Data on intensity or duration of tobacco use were not available, but this was likely a major contributor to the high incidence of lung cancer.

In the calendar years of our study, no decreases in incidence were apparent for KS or NADCs. Given this consistent cancer burden, the lack of validation of most cancer treatment regimens in the HIV population, and the high cancer risk compared to the general population, a focus on increased screening and prevention efforts is warranted.

## Competing interests

The authors have no competing interests to declare.

## Authors’ contributions

DD, EY, and SN led the conception and design of the study, contributed to data acquisition and interpretation, prepared the initial draft of the manuscript, had full access to the data in the study, and take final responsibility for the decision to submit for publication. KT, BD and JJE substantively contributed to the study design, data interpretation and provided critical revision of the manuscript. All authors approved the final version of the manuscript.
